# Quantitative Relationship Between Cumulative Risk Alleles Based on Genome-Wide Association Studies and Type 2 Diabetes Mellitus: A Systematic Review and Meta-analysis

**DOI:** 10.2188/jea.JE20160151

**Published:** 2018-01-05

**Authors:** Satoru Kodama, Kazuya Fujihara, Hajime Ishiguro, Chika Horikawa, Nobumasa Ohara, Yoko Yachi, Shiro Tanaka, Hitoshi Shimano, Kiminori Kato, Osamu Hanyu, Hirohito Sone

**Affiliations:** 1 Department of Laboratory Medicine and Clinical Epidemiology for Prevention of Noncommunicable Niigata University Graduate School of Medical and Dental Sciences, Niigata, Japan; 2 Department of Hematology, Endocrinology and Metabolism, Niigata University Faculty of Medicine, Niigata, Japan; 3 Department of Health and Nutrition, Faculty of Human Life Studies, University of Niigata Prefecture, Niigata, Japan; 4 Department of Administrative Dietetics, Faculty of Health and Nutrition, Yamanashi Gakuin University, Yamanashi, Japan; 5 Department of Clinical Trial, Design & Management, Translational Research Center, Kyoto University Hospital, Japan; 6 Department of Internal Medicine, University of Tsukuba Institute of Clinical Medicine, Ibaraki, Japan

**Keywords:** genome-wide association studies, risk allele, type 2 diabetes mellitus, meta-analysis

## Abstract

Many epidemiological studies have assessed the genetic risk of having undiagnosed or of developing type 2 diabetes mellitus (T2DM) using several single nucleotide polymorphisms (SNPs) based on findings of genome-wide association studies (GWAS). However, the quantitative association of cumulative risk alleles (RAs) of such SNPs with T2DM risk has been unclear. The aim of this meta-analysis is to review the strength of the association between cumulative RAs and T2DM risk. Systematic literature searches were conducted for cross-sectional or longitudinal studies that examined odds ratios (ORs) for T2DM in relation to genetic profiles. Logarithm of the estimated OR (log OR) of T2DM for 1 increment in RAs carried (1-ΔRA) in each study was pooled using a random-effects model. There were 46 eligible studies that included 74,880 cases among 249,365 participants. In 32 studies with a cross-sectional design, the pooled OR for T2DM morbidity for 1-ΔRA was 1.16 (95% confidence interval [CI], 1.13–1.19). In 15 studies that had a longitudinal design, the OR for incident T2DM was 1.10 (95% CI, 1.08–1.13). There was large heterogeneity in the magnitude of log OR (*P* < 0.001 for both cross-sectional studies and longitudinal studies). The top 10 commonly used genes significantly explained the variance in the log OR (*P* = 0.04 for cross-sectional studies; *P* = 0.006 for longitudinal studies). The current meta-analysis indicated that carrying 1-ΔRA in T2DM-associated SNPs was associated with a modest risk of prevalent or incident T2DM, although the heterogeneity in the used genes among studies requires us to interpret the results with caution.

## INTRODUCTION

Intensive lifestyle interventions (eg, promoting increased physical activity and weight loss) can be effective in decreasing the incidence of type 2 diabetes mellitus (T2DM).^1^ However, healthcare resources are limited, and participants in interventions to prevent diabetes should be prioritized. Identification of individuals at high risk of T2DM could facilitate the targeting of prevention efforts to those who could benefit from them and reduce the cost of preventing T2DM.

Predicting T2DM in healthy individuals has been attempted using a diabetes risk score that is derived from common clinical information, such as adiposity, blood pressure, and family history of T2DM. However, using the risk score is inevitably limited in predicting T2DM because T2DM has a strong genetic basis; concordance of T2DM is about 70% for monozygotic twins, compared to about 20–30% for dizygotic twins.^2^

Limitations in predicting T2DM have driven researchers to employ genetic risk assessments. Moreover, unlike clinical markers, genetic markers do not change with time, so they possess the advantage of identifying high-risk individuals long before disease onset, which could enable early interventions for preventing T2DM. Conventionally, family-based linkage studies have played an important role in identifying genes having a large effect in monogenic disorders, such as maturity-onset diabetes of the young.^3^ However, linkage studies have low power for polygenic diseases that are influenced by multiple genes, as is the case with the majority of those with T2DM. Therefore, using monogenic mutations would have very limited value for predicting risk of disease in the general population because of their low frequency.

Genome-wide association studies (GWAS) capture the great majority of common genetic differences among individuals and relate them to health and diseases.^4^ The GWAS potentially represent a powerful new tool for identifying genes that influence common diseases. Recently, GWAS identified an increasing number of loci associated with susceptibility to T2DM. However, each risk allele (RA) of single nucleotide polymorphisms (SNPs) at these loci has a modest effect size.^5^ Therefore, a combination of several SNPs is required to substantially influence T2DM risk. A genetic risk score potentially has the ability to predict disease risk as a function of the combined effects of SNPs. The most typical approach to produce the genetic risk score is to count the total number of RAs at each T2DM-associated SNP identified by GWAS. Because the number of RAs carried is consequently a quantifiable variable, it can be potentially used for clinical risk assessment, similar to measurement of body mass index (BMI). Recently, an increasing number of epidemiological studies have investigated the effect of cumulative RAs on T2DM risk. However, the quantitative association between cumulative RAs and T2DM risk has not been established, while the association of BMI with T2DM risk was quantified using meta-analysis.^6^ The aim of this meta-analysis is to comprehensively estimate the strength of the association between cumulative RAs and T2DM risk, including exploration of differences in the magnitude of T2DM risk according to several study characteristics.

## MATERIALS AND METHODS

### Literature searches

Electronic literature search using EMBASE and MEDLINE (up to 2015) was conducted for studies that quantified the genetic risk of T2DM. This search was limited to articles published after the International HapMap Project had identified a majority of the common SNPs examined by GWAS in 2003.^7^ Manual searches were added using the reference list of each included study.

### Inclusion criteria

Study keywords were thesaurus terms related to genetic profiles and T2DM (Table 1). Inclusion criteria were: 1) cross-sectional design (for assessing the possibility of having undiagnosed T2DM) or longitudinal design (for assessing the risk of developing T2DM); 2) total number of RAs carried calculated by summing the number of RAs in each SNP according to an additive model (ie, 0, 1, and 2 were assigned for homozygosity for a non-RA, heterozygosity for an RA, and homozygosity for an RA, respectively) were analyzed as an exposure; 3) two or more SNPs were combined for assessing genetic risk; and 4) the odds ratio (OR) for each increment in the number of RAs carried (1-ΔRA) and its corresponding standard error (SE) (SE is usually provided for the natural logarithm of OR [log OR] rather than for the OR) were presented or could be estimated. Exceptionally, studies that used the dominant model (ie, counting 1 for homozygosity or heterozygosity for having an RA and 0 for having no RA) were also considered.

**Table 1.** Study keywords for this meta-analysis

Using EMBASE
Terms related to thesaurus
#1 [related to genetic backgrounds]
“genetic variability” OR “genetic polymorphism” OR “single nucleotide polymorphism [Exp]” OR “genetic association” OR “genotyping” OR “genetic susceptibility” OR “genetic resistance” OR “genetic predisposition”
#2 [related to type 2 diabetes mellitus]
“non insulin dependent diabetes mellitus -- epidemiology” OR “diabetes mellitus -- epidemiology”
#3 #1 AND #2
Using MEDLINE
#4 [related to genetic backgrounds]
“Genome-Wide Association Study” OR “Genetic Association Studies” OR “Polymorphism, Single Nucleotide” OR “Genetic Variation [Exp]” OR “Polymorphism, Genetic [Exp]” OR “Genotype” OR “Genetic Predisposition to Disease”
#5 [related to type 2 diabetes mellitus]
“Diabetes Mellitus, Type 2 -- epidemiology” OR “Diabetes Mellitus, Type 2 -- genetics” OR “Diabetes Mellitus -- genetics” OR “Diabetes Mellitus -- epidemiology”
#6 #4 AND #5
Combination of EMBASE and MEDLINE
#7 #3 OR #6

[Exp] indicates automatic inclusion of all the narrower terms under the specified descriptor in the thesaurus hierarchy.

Using the connector “--” limits a descriptor term to a Subheading appearing after the connector.

Haplotypes into which alignments of multiple SNPs were classified were regarded as one unit because these SNPs could not be separated into individual SNPs. Therefore, studies that examined the association between several haplotypes and T2DM risk were excluded, even if two or more SNPs were used. Due to the necessity of maintaining homogeneity across studies as closely as possible, the OR needed to be adjusted for at least two of the following three covariates: age, gender, and BMI. For the same reason, studies were excluded if they examined T2DM risk in relation to a weighted risk score that was produced by summing the number of RAs multiplied by the SNP-specific effect size. In longitudinal studies, if the risk measure was expressed as a relative risk (RR) rather than OR, we included such studies on the condition that the incidence rate in the study population was reported. In that case, the RR was converted into an OR using the following formula: $\mathrm{OR}=\frac{\mathrm{RR}\times (1-{\text{P}}_{\text{o}})}{1-{\text{P}}_{\text{o}}\times \mathrm{RR}}$, in which the incidence rate was imputed to P_o_.^8^ Also, as the SE corresponding to the natural logarithm of RR (log RR) was converted into the SE corresponding to log OR, the Miettinen test-based approach was applied: $\text{SE}(\text{log}\mathrm{OR})=\text{SE}(\text{log}\mathrm{RR})\times \sqrt{\frac{\text{log}\mathrm{OR}}{\text{log}\mathrm{RR}}}$.^9^

### Data extraction

For each included study, two authors (S.K. and H.So.) extracted the following information relevant to study characteristics: country, ethnic group (if information was presented), design (cross-sectional or longitudinal), observational periods (in case of a longitudinal study), number of participants and cases, mean age, proportion of men and women, mean BMI, study-specific covariates, criteria of T2DM cases and non-cases, and used SNPs. Inconsistencies were resolved via discussion. Study quality was assessed by 10 basic questions that should be answered in the affirmative to indicate a reliable report (Table 2).^4^ One point was awarded to a study for each “Yes” answer, with a maximum score of 10.

**Table 2.** Assessment of study quality using 10 basic questions about genome-wide association studies

First author	Year	Quality Score	Q.1	Q.2	Q.3	Q.4	Q.5	Q.6	Q.7	Q.8	Q.9	Q.10
Oian^18^	2015	8	Yes	Yes	Yes	Yes	Yes	Yes	No	Yes	Yes	No
Talmud^17^	2015	6	Yes	Yes	No	Yes	Yes	Yes	Yes	No	No	No
Chen^21^	2014	5	No	No	Yes	Yes	Yes	Yes	Yes	No	No	No
Langenberg^20^	2014	5	No	Yes	No	Yes	Yes	Yes	Yes	No	No	No
Villegas^19^	2014	3	No	No	No	No	Yes	Yes	Yes	No	No	No
Anand^26^	2013	3	No	Yes	No	Yes	No	Yes	No	No	No	No
Kalnina^27^	2013	5	Yes	No	No	Yes	Yes	No	Yes	No	No	Yes
Imamura^25^	2013	5	Yes	No	Yes	Yes	Yes	Yes	No	No	No	No
Peters^28^	2013	4	No	No	No	No	Yes	Yes	Yes	No	No	Yes
Ramya^24^	2013	4	Yes	No	No	No	Yes	Yes	Yes	No	No	Yes
Robiou-du-Pont^23^	2013	5	Yes	No	Yes	Yes	No	Yes	Yes	No	No	No
Tam^22^	2013	4	Yes	No	Yes	Yes	No	Yes	No	No	No	No
Cauchi^29^	2012	3	Yes	No	No	Yes	No	Yes	No	No	No	No
Cooke^30^	2012	5	Yes	No	Yes	Yes	Yes	Yes	No	No	No	No
Gamboa-Meiendez^31^	2012	3	Yes	No	No	Yes	No	Yes	No	No	No	No
Iwata^32^	2012	8	Yes	No	Yes	Yes	Yes	Yes	Yes	Yes	Yes	No
Long^33^	2012	5	Yes	No	Yes	Yes	Yes	Yes	No	No	No	No
Vassy^34^	2012	3	Yes	Yes	No	No	No	Yes	No	No	No	No
Vassy^35^	2012	3	Yes	Yes	No	No	Yes	No	No	No	No	No
Villegas^36^	2012	5	Yes	No	Yes	Yes	Yes	Yes	No	No	No	No
Yamakawa-Kobayashi^37^	2012	3	Yes	No	No	Yes	No	Yes	No	No	No	No
Li^38^	2011	3	No	Yes	No	Yes	No	Yes	No	No	No	No
Martinez-Gomez^39^	2011	6	Yes	No	No	Yes	Yes	No	Yes	Yes	Yes	No
Rees^40^	2011	4	Yes	No	Yes	Yes	No	Yes	No	No	No	No
Tabara^41^	2011	3	Yes	Yes	No	No	No	Yes	No	No	No	No
Uusitupa^42^	2011	4	Yes	Yes	No	Yes	No	Yes	No	No	No	No
Fontaine-Bisson^43^	2010	4	Yes	No	No	Yes	No	Yes	No	No	No	Yes
Qi^44^	2010	3	Yes	No	No	Yes	No	Yes	No	No	No	No
Rotger^45^	2010	1	No	No	No	No	No	Yes	No	No	No	No
Wang^46^	2010	3	Yes	No	No	Yes	No	Yes	No	No	No	No
Waters^47^	2010	2	No	No	Yes	No	No	Yes	No	No	No	No
Xu^48^	2010	7	Yes	Yes	Yes	Yes	Yes	Yes	Yes	No	No	No
Cornelis^49^	2009	5	No	Yes	Yes	Yes	Yes	Yes	No	No	No	No
Hu^50^	2009	6	Yes	No	Yes	Yes	Yes	Yes	Yes	No	No	No
Lin^51^	2009	3	Yes	No	No	Yes	No	Yes	No	No	No	No
Miyake^52^	2009	6	No	No	Yes	No	Yes	Yes	Yes	Yes	Yes	No
Nordman^53^	2009	3	Yes	No	No	Yes	No	Yes	No	No	No	No
Rong^54^	2009	2	Yes	No	No	No	No	Yes	No	No	No	No
Schulze^55^	2009	2	No	No	No	Yes	No	Yes	No	No	No	No
Cauchi^56^	2008	7	Yes	No	Yes	Yes	Yes	Yes	Yes	Yes	No	No
Lyssenko^57^	2008	7	Yes	No	Yes	Yes	Yes	Yes	No	Yes	Yes	No
Meigs^58^	2008	4	Yes	No	No	Yes	Yes	Yes	No	No	No	No
Vaxillaire^59^	2008	5	Yes	No	No	Yes	Yes	Yes	Yes	No	No	No
Scott^60^	2006	4	Yes	No	No	Yes	Yes	Yes	No	No	No	No
Hansen^61^	2005	5	Yes	No	No	Yes	Yes	Yes	No	No	No	Yes
Zacharova^62^	2005	4	Yes	Yes	No	No	No	Yes	No	No	No	Yes

Each question used for assessment of study quality is given below.^4^ If the answer is “Yes” to a question, a study could be awarded 1 point. Full score is 10.

Q.1. Are the cases defined clearly and reliably so that they can be compared with patients typically seen in clinical practice?

Q.2. Are case and control participants demonstrated to be comparable to each other for important characteristics that might also be related to genetic variation and to the disease? In longitudinal studies, the answer was considered to be “Yes” if the diabetes risk was adjusted for age, gender, and obesity index.

Q.3. Was the study of sufficient size to detect modest odds ratios or relative risks (1.3–1.5)?

(At least 1,500 cases are needed to obtain 90% statistical power for detecting a 30% allelle with a 1.5 odds ratio at *P* < 10^−8^ of a significant level.^*^)

Q.4. Was the genotyping platform of sufficient density to capture a large proportion of the variation in the population studied?

Q.5. Were appropriate quality control measures applied to genotyping assays, including visual inspection of cluster plots and replication on an independent genotyping platform?

(The concordance rate needed to be presented by using a duplicate sample if the study met this criterion.)

Q.6. Did the study reliably detect associations with previously reported and replicated variants (known positives)?

Q.7. Were stringent corrections applied for the many thousands of statistical tests performed in defining the *P* value for significant associations?

Q.8. Were the results replicated in independent population samples?

Q.9. Were the replication samples comparable in geographic origin and phenotype definition, and if not, did the differences extend the applicability of the findings?

Q.10. Was evidence provided for a functional role for the gene polymorphism identified?

^*^Altshuler D, Daly MJ, Lander ES. Genetic mapping in human disease. Science 2008;322:881–8.

If a study presented several ORs for T2DM with different levels of adjustment, the most completely adjusted OR was chosen. If a study presented several ORs with different combinations of SNPs used for T2DM screening, the OR that had the greatest effect size was chosen. In addition, if a study performed both overall and subgroup analyses, we chose the data based on subgroup analyses if characteristics, such as mean age, proportion of men, and mean BMI, in each subgroup were described; otherwise, data based on an overall analysis were chosen.

### Data synthesis

Most of the included studies did not directly present data on the OR for the increment in the number of RAs carried. To estimate the OR, the log ORs in several genetic risk groups in the individual study were regressed to their corresponding mean number of RAs carried.^10^ If data on the mean number of RAs carried could not be directly extracted, the midpoint value of the upper and lower boundaries of the number of RAs carried was used for intermediate categories. In other categories (ie, highest or lowest category), we assumed that the frequency distribution of the number of RAs carried was normal and regressed the number of RAs carried to its corresponding Z-value for the rank percentile in the upper and lower boundary in each intermediate category and extrapolated the regression line into the highest and lowest categories. This regression is called generalized least squares for trend estimation (GLST), for which a program was developed by Orsini et al.^11^ This program can calculate a weighted linear regression of log OR across categories of the number of RAs carried with consideration of the covariance among the log ORs as long as data on the total number of participants and cases are provided.

Cross-sectional studies and longitudinal studies were analyzed separately. In each study, the OR of T2DM for 1-ΔRA was transformed into log OR because its corresponding SE is provided for log OR rather than for the OR. The log OR was pooled with a random-effects model^12^ followed by exponentiation of the pooled log OR to obtain the OR of interest. Study heterogeneity was assessed by Q-statistics or I-squared overall and within each strata after stratification.^13^

Publication bias was assessed by two formal statistical tests: Begg’s rank correlation test^14^ and Egger’s regression asymmetry test.^15^ For statistically suspected publication bias, the trim and fill method was adopted to adjust the pooled T2DM risk, assuming that the asymmetry of the funnel plot is entirely due to publication bias. This method includes detection of unpublished studies that distorted the funnel plot, filling the results of these hypothetical studies to recover the symmetry of the funnel plot, and recalculation of the pooled effect size as if these studies had actually existed.^16^

### Sensitivity analysis

The pooled OR was estimated for subgroups after stratification on the basis of the following pre-specified study characteristics: number of SNPs (<10 or ≥10), mean age (<55 years or ≥55 years in cross-sectional studies; <50 years or ≥50 years in longitudinal studies), proportion of men (<50% or ≥50%), country where the study was conducted (Western or non-Western), ethnic group (Asian or non-Asian), mean BMI (<27 kg/m^2^ or ≥27 kg/m^2^ in cross-sectional studies; <25 kg/m^2^ or ≥25 kg/m^2^ in longitudinal studies), whether the OR was or was not adjusted for BMI, whether the oral glucose tolerance test (OGTT) was thoroughly performed for detecting any cases of T2DM, and whether subjects with impaired glucose tolerance (IGT) or impaired fasting glucose (IFG) were excluded from non-T2DM cases. The cut-off values for mean age and BMI used in the stratified analyses corresponded to these median values in cross-sectional and longitudinal studies (median age: 55.7 years for cross-sectional studies and 50.6 years for longitudinal studies; median BMI: 26.7 kg/m^2^ for cross-sectional studies and 25.9 kg/m^2^ for longitudinal studies). Univariate log-linear meta-regression analysis was used to test the differences in the magnitude of the OR between strata.

Multivariate log-linear meta-regression analyses were added using several study characteristics simultaneously as explanatory variables. In these regression analyses, log OR of T2DM for 1-ΔRA was used as an objective variable. The T2DM-susceptible genes that were used in each study were inconsistent. Therefore, we added a meta-regression analysis of whether or not each of these genes was examined. This analysis was limited to the top 10 of the commonly used genes in this meta-analysis in order to maintain statistical power. All analyses were based on statistical software STATA version 14 (STATA Corp., College Station, TX, USA). The meta-regression analyses were performed by installing the “metareg” command into STATA. We also installed the “glst” command to estimate the OR for carrying 1-ΔRA using GLST.

## RESULTS

### Eligible studies

**Figure 1.**
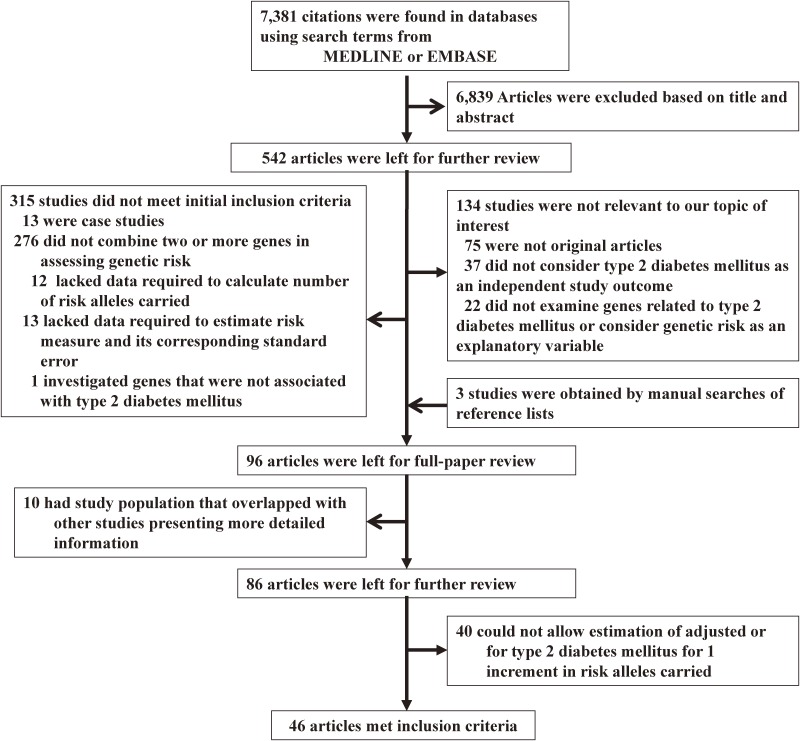
Flowchart of search for eligible studies

Figure 1 is a flow chart showing the procedure for identifying studies that met the initial inclusion criteria. Of 7,381 studies retrieved through the electronic literature search, 96 investigated the genetic risk for T2DM combining two or more SNPs. After 10 studies in which the study population overlapped with other studies were eliminated, there remained 86 studies for further review. Forty studies did not allow estimation of the adjusted OR for T2DM for one increment in risk alleles carried. Finally, 46 original studies^17^^–^^62^ in which the OR for T2DM for 1-ΔRA was presented or could be estimated, including 75,651 cases among 249,365 participants, were eligible. Twenty-eight studies^17^^,^^19^^,^^20^^,^^23^^,^^26^^,^^27^^,^^29^^,^^30^^,^^33^^–^^35^^,^^38^^,^^40^^,^^42^^,^^43^^,^^45^^–^^47^^,^^49^^,^^51^^,^^53^^,^^55^^–^^60^^,^^62^ analyzed T2DM risk in Western countries. Table 3 shows characteristics of the 46 eligible studies. Most studies were not considered to have targeted exclusively a single ethnicity, and only 5 studies^19^^,^^26^^,^^39^^,^^47^^,^^57^ performed analyses by ethnicity. Only 2 studies^49^^,^^62^ analyzed men and women separately, and all but 3 studies^37^^,^^46^^,^^53^ included both genders. One study^48^ analyzed T2DM risk from both cross-sectional and longitudinal perspectives.

**Table 3.** Characteristics of each eligible study analyzed in this meta-analysis

First author	Year	Country	Design	Follow-up duration^a^ (years)	Subgroup	Number of participants	Number of cases	Mean age	% men	BMI
Oian^18^	2015	China	C			6,063	2,853	57.3	37.5%	23.5
Talmud^17^	2015	UK	L	10		13,294	804	N/A	59.5%	N/A
Chen^21^	2014	Singapore	C			4,677	2,338	55.2	47.1%	23.6
Langenberg^20^	2014	UK	L	8.9		18,890	8,245	52.3	38.0%	26.7
Villegas^19^	2014	USA	C		Non-Hispanic Whites	6,377	545	52.8	45.0%	27.4
					Non-Hispanic Blacks	3,054	337	43.7	44.0%	28.9
					Mexican Americans	3,621	455	43.6	49.0%	28.2
Anand^26^	2013	Canada	L	3.3	European	5,449	586	55.0	39.2%	30.4
					South African	2,268	194	44.9	51.6%	26.4
					Latinos	2,815	218	52.6	33.2%	30.8
Imamura^25^	2013	Japan	C			4,399	2,613	58.1	45.2%	23.8
Kalnina^27^	2013	Latvia	C			2,047	981	56.7	32.1%	29.7
Peters^28^	2013	Australia	C			3,322	967	55.7	47.4%	26.9
Ramya^24^	2013	India	C			1,957	940	45.8	44.3%	24.3
Robiou-du-Pont^23^	2013	France	C			5,162	2,077	54.5	43.5%	26.7
Tam^22^	2013	China	C			8,451	5,882	52.2	46.1%	24.1
Cauchi^29^	2012	France	C			2,248	1,193	56.1	32.5%	28.1
Cooke^30^	2012	USA	C			4,045	2,652	55.7	41.9%	30.9
Gamboa-Meiendez^31^	2012	Mexico	C			2,017	1,027	53.5	37.6%	28.6
Iwata^32^	2012	Japan	C		Other than Tokyo University	1,487	724	68.8	54.5%	23.6
					Tokyo University	2,041	1,182	67.1	53.2%	24.0
Long^33^	2012	USA	C			4,288	1,554	56.8	30.7%	31.5
Vassy^34^	2012	USA	L	23.9		2,439	215	25.1	44.0%	24.3
Vassy^35^	2012	USA	L	26.9		1,030	90	14.4	44.7%	20.7
Villegas^36^	2012	China	C			6,001	2,679	55.6	23.8%	26.4
Yamakawa-Kobayashi^37^	2012	Japan	C			750	333	54.0	100%	23.9
Li^38^	2011	UK	L	12.9		21,157	729	58.9	49.8%	26.5
Martinez-Gomez^39^	2011	Mexico	C		Guerrero	400	200	50.7	36.0%	28.2
					Mexico	1,065	546	48.6	50.5%	28.4
Rees^40^	2011	UK	C			3,262	1,659	55.8	49.4%	26.3
Tabara^41^	2011	Japan	L	9.4		1,824	95	62.0	54.4%	23.5
Uusitupa^42^	2011	Finland	L	6.2		522	185	55.4	32.8%	31.2
Fontaine-Bisson^43^	2010	Sweden	C			2,751	1,327	53.3	54.2%	27.6
Qi^44^	2010	China	C			2,332	424	58.8	42.8%	24.2
Rotger^45^	2010	Swiss	C			644	94	39.9	79.5%	23.2
Wang^46^	2010	Finland	C			7,232	518	57.7	100%	27.0
Waters^47^	2010	USA	C		African-Americans	2,546	1,077	60.2	40.9%	28.6
					Native Hawaiians	1,559	576	55.6	48.2%	28.7
					European-Americans	1,539	533	57.6	51.5%	26.6
					Latinos	4,404	2,220	59.3	48.0%	27.7
					Japanese	3,497	1,736	59.2	56.2%	25.2
Xu^48^	2010	China	C			5,512	1,825	61.1	40.9%	25.2
			L	3.5		734	67	60.0	39.0%	24.8
Cornelis^49^	2009	USA	L	10		6,310	2,809	48.5	40.2%	25.9
Hu^50^	2009	China	C			3,634	1,849	59.3	46.9%	23.8
Lin^51^	2009	USA	C			5,360	356	53.3	47.4%	25.8
Miyake^52^	2009	Japan	C			4,678	2,316	64.4	52.1%	23.5
Nordman^53^	2009	Sweden	C			771	243	52.3	100%	29.5
Rong^54^	2009	Iadia	C			2,745	1,161	33.7	N/A	36.9
Schulze^55^	2009	Germany	L	7		2,541	579	50.6	51.1%	26.9
Cauchi^56^	2008	France	C			8,827	4,232	58.6	41.9%	26.7
Lyssenko^57^	2008	Sweden	L	23.5	Malmo	16,061	2,063	45.5	51.7%	24.3
					Botnia	2,770	138	44.9	64.9%	25.6
Meigs^58^	2008	USA	L	28		2,434	255	35.0	45.5%	24.9
Vaxillaire^59^	2008	France	L	9		3,442	292	47.7	46.8%	24.3
Scott^60^	2006	USA	C			2,104	1,151	64.2	44.3%	28.4
Hansen^61^	2005	Denmark	C			5,897	1,164	48.4	55.8%	26.3
Zacharova^62^	2005	Finland	L	3.3		223	102	54.7	49.5%	30.9^**^

BMI, body mass index; C, cross-sectional; L, longitudinal.

^a^In study using longitudinal design.

Table 4 summarizes the criteria for T2DM cases and non-cases in each study. OGTT was used for any participant in whom T2DM was undiagnosed using other methods in 20 studies.^22^^,^^24^^,^^26^^,^^30^^,^^37^^,^^40^^,^^43^^,^^45^^,^^46^^,^^48^^–^^50^^,^^53^^,^^54^^,^^57^^,^^58^^,^^60^^–^^63^ Among cross-sectional studies, 7 studies^24^^,^^48^^,^^50^^,^^53^^,^^54^^,^^60^^,^^61^ and 13 studies^18^^,^^29^^,^^31^^,^^33^^,^^36^^,^^37^^,^^44^^,^^48^^,^^50^^,^^53^^,^^56^^,^^60^^,^^64^ excluded participants with IGT and IFG, respectively, from non-T2DM cases. Table 5 shows details of covariates that each study arbitrarily considered. Study-specific covariates were heterogeneous among studies. However, T2DM risk was adjusted for BMI in most studies (37 studies).^17^^–^^20^^,^^22^^–^^28^^,^^31^^–^^38^^,^^40^^–^^42^^,^^44^^–^^56^^,^^59^^,^^62^

**Table 4.** Criteria for cases and non-cases in each included study

First author	Design	Criteria	
		Cases	Non-cases
Oian^18^	C	FPG ≥ 7.0 mmol/l	FPG < 5.6 mmol/l
Talmud^17^	L	self-report or FPG ≥ 7.0 mmol/l	FPG < 7.0 mmol/l
Chen^21^	C	interview or A1C > 6.0%	A1C ≤ 6.0%
Langenberg^20^	L	self-report, registry, medical record	unclear
Villegas^19^	C	questionnaire	questionnaire
Anand^26^	L	FPG ≥ 7.0 mmol/l or 2hPG ≥ 11.1 mmol/l	IFG or IGT
Kalnina^27^	C	FPG ≥ 7.0 mmol/l or 2hPG ≥ 11.1 mmol/l	FPG < 7.0 mmol/l, 2hPG < 11.1 mmol/l
Imamura^25^	C	registry	registry
Peters^28^	C	unclear	unclear
Ramya^24^	C	2hPG ≥ 11.1 mmol/l	2hPG < 7.8 mmol/l
Robiou-du-Pont^23^	C	FPG ≥ 7.0 mmol/l	FPG < 7.0 mmol/l
Tam^22^	C	FPG ≥ 7.0 mmol/l or 2hPG ≥ 11.1 mmol/l	unclear
Cauchi^29^	C	FPG ≥ 7.0 mmol/l	FPG < 5.6 mmol/l
Cooke^30^	C	FPG ≥ 7.0 mmol/l or 2hPG ≥ 11.1 mmol/l	FPG < 7.0 mmol/l, 2hPG < 11.1 mmol/l
Gamboa-Meiendez^31^	C	casual PG ≥ 11.1 mmol/l or FPG ≥ 7.0 mmol/l	FPG < 5.6 mmol/l, no FH of DM
Iwata^32^	C	unclear	A1C < 6.0%
Long^33^	C	casual PG ≥ 11.1 mmol/l or A1C ≥ 6.5%	FPG < 6.1 mmol/l, A1C < 6.0%
Vassy^34^	L	FPG ≥ 7.0 mmol/l	FPG < 7.0 mmol/l
Vassy^35^	L	FPG ≥ 7.0 mmol/l	FPG < 7.0 mmol/l
Villegas^36^	C	FPG ≥ 7.0 mmol/l	FPG < 5.6 mmol/l, A1C < 6.1%
Yamakawa-Kobayashi^37^	C	FPG ≥ 7.0 mmol/l or 2hPG ≥ 11.1 mmol/l	FPG < 5.6 mmol/l, A1C < 5.8%
Li^38^	L	FPG ≥ 7.0 mmol/l	FPG < 7.0 mol/l
Martinez-Gomez^39^	C	FPG ≥ 7.0 mmol/l	FPG < 7.0 mol/l
Rees^40^	C	FPG ≥ 7.0 mmol/l or 2hPG ≥ 11.1 mmol/l	FPG < 5.6 mmol/l or (FPG < 6.1 mmol/l, 2hPG < 7.8 mmol/l) or casual PG < 6.7 mmol/l
Tabara^41^	L	FPG ≥ 7.0 mmol/l or 2hPG ≥ 11.1 mmol/l	FPG < 7.0 mmol/l, 2hPG < 11.1 mmol/l
Uusitupa^42^	L	FPG ≥ 7.8 mmol/l or 2hPG ≥ 11.1 mmol/l	FPG < 7.8 mmol/l, 2hPG < 11.1 mmol/l
Fontaine-Bisson^43^	C	FPG ≥ 7.0 mmol/l or 2hPG ≥ 11.1 mmol/l	FPG < 7.0 mmol/l, 2hPG < 11.1 mmol/l
Qi^44^	C	FPG ≥ 7.0 mmol/l	FPG < 5.6 mmol/l
Rotger^45^	C	casual PG ≥ 11.1 mmol/l or FPG ≥ 7.0 mmol/l	casual PG < 11.1 mmol/l, FPG < 7.0 mmol/l
Wang^46^	C	FPG ≥ 7.0 mmol/l or 2hPG ≥ 11.1 mmol/l	FPG < 7.0 mmol/l, 2hPG < 11.1 mmol/l
Waters^47^	C	unclear	unclear
Xu^48^	C	FPG ≥ 7.0 mmol/l or 2hPG ≥ 11.1 mmol/l	FPG < 6.1 mmol/l, 2hPG < 7.8 mmol/l
	L	FPG ≥ 7.0 mmol/l or 2hPG ≥ 11.1 mmol/l	FPG < 7.0 mmol/l, 2hPG < 11.1 mmol/l
Cornelis^49^	L	FPG ≥ 7.8 mmol/l or 2hPG ≥ 11.1 mmol/l	FPG < 7.8 mmol/l, 2hPG < 11.1 mmol/l
Hu^50^	C	FPG ≥ 7.0 mmol/l or 2hPG ≥ 11.1 mmol/l	FPG < 6.1 mmol/l, 2hPG < 7.8 mmol/l
Lin^51^	C	FPG ≥ 7.0 mmol/l	FPG < 7.0 mmol/l
Miyake^52^	C	physician diagnosis for Japanese, registry for Chinese	A1C < 5.6% for Japanese, FPG < 6.1 mmol/l for Chinese
Nordman^53^	C	FPG ≥ 7.0 mmol/l or 2hPG ≥ 11.1 mmol/l	FPG < 6.1 mmol/l, 2hPG < 7.8 mmol/l
Rong^54^	C	FPG ≥ 7.8 mmol/l or 2hPG ≥ 11.1 mmol/l	FPG < 7.8 mmol/l, 2hPG < 11.1 mmol/l
Schulze^55^	L	questionnaire	questionnaire
Cauchi^56^	C	casual PG ≥ 11.1 mmol/l or FPG ≥ 7.8 mmol/l	FPG < 6.1 mmol/l, no FH of DM
Lyssenko^57^	L	FPG ≥ 7.0 mmol/l or 2hPG ≥ 11.1 mmol/l	FPG < 7.0 mmol/l, 2hPG < 11.1 mmol/l
Meigs^58^	L	FPG ≥ 7.8 mmol/l or 2hPG ≥ 11.1 mmol/l	FPG < 7.0 mmol/l, 2hPG < 11.1 mmol/l
Vaxillaire^59^	L	FPG ≥ 7.0 mmol/l	FPG < 7.0 mmol/l
Scott^60^	C	FPG ≥ 7.8 mmol/l or 2hPG ≥ 11.1 mmol/l	FPG < 6.1 mmol/l, 2hPG < 7.8 mmol/l
Hansen^61^	C	FPG ≥ 7.0 mmol/l or 2hPG ≥ 11.1 mmol/l	FPG < 6.1 mmol/l, 2hPG < 7.8 mmol/l
Zacharova^62^	L	FPG ≥ 7.8 mmol/l or 2hPG ≥ 11.1 mmol/l	7.8 mmol/l = < 2hPG < 11.1 mmol/l, FPG < 7.8 mmol/l

2hPG, 2-hour post glucose concentration; A1C, hemoglobin A_1C_; ADA, American Diabetes Association criteria for diabetes; FH of DM, family history of diabetes mellitus; FPG, fasting plasma glucose; IFG, impaired fasting glucose; IGT, impaired glucose tolerance.

**Table 5.** Details of covariates considered in each included study

First author	Year	Covariates
Oian^18^	2015	age, gender, BMI
Talmud^17^	2015	age, gender, BMI, BP, HDL, TG
Chen^21^	2014	age, gender, dialect, global ancestry
Langenberg^20^	2014	age, gender, BMI
Villegas^19^	2014	age, gender, BMI
Anand^26^	2013	age, gender, BMI, WC, FHDM, smoking, PA, Apo-A, Apo-B, HT
Imamura^25^	2013	age, gender, BMI
Kalnina^27^	2013	age, gender, BMI
Peters^28^	2013	age, gender, BMI, adiponectine
Ramya^24^	2013	age, gender, BMI
Robiou-du-Pont^23^	2013	age, gender, BMI
Tam^22^	2013	age, gender, BMI
Cauchi^29^	2012	age, gender
Cooke^30^	2012	age, gender
Gamboa-Meiendez^31^	2012	age, gender, BMI
Iwata^32^	2012	age, gender, BMI
Long^33^	2012	age, gender, BMI
Vassy^34^	2012	age, gender, race, FH of DM, BMI, FPG, HDL, TG, PA, smoking, alcohol
Vassy^35^	2012	age, gender, race, FH, BMI, MAP, FPG, HDL, TG
Villegas^36^	2012	age, gender, BMI
Yamakawa-Kobayashi^37^	2012	age, gender, BMI
Li^38^	2011	age, gender, BMI
Martinez-Gomez^39^	2011	age, gender
Rees^40^	2011	age, gender, BMI
Tabara^41^	2011	age, gender, BMI
Uusitupa^42^	2011	age, gender, BMI, BMI change, FH, intervention, FPG, 2hPG, FPG change, 2hPG change, insulin, insulin change, 2h-insulin, 2h-insulin change
Fontaine-Bisson^43^	2010	age, gender
Qi^44^	2010	age, gender, BMI
Rotger^45^	2010	age, gender, BMI, treatment for HIV, CD4+, HDL, TG
Wang^46^	2010	## FINDRISC score, TG, HDL, ALT, adiponectine
Waters^47^	2010	age, gender, BMI
Xu^48^	2010	age, gender, BMI, FH of DM, smoking, alcohol
Cornelis^49^	2009	age, gender, BMI, FH of DM, smoking, alcohol, PA
Hu^50^	2009	age, gender, BMI
Lin^51^	2009	age, BMI, FH of DM, TG/HDL, PA
Miyake^52^	2009	age, gender, BMI
Nordman^53^	2009	age, (gender), BMI, SBP, DBP
Rong^54^	2009	age, gender, BMI
Schulze^55^	2009	DRS score^a^, HDL, TG, ALT
Cauchi^56^	2008	age, gender, BMI
Lyssenko^57^	2008	age, gender
Meigs^58^	2008	age, gender
Vaxillaire^59^	2008	age, gender, BMI
Scott^60^	2006	age, gender, birth province
Hansen^61^	2005	age, gender
Zacharova^62^	2005	age, (gender), BW, BW change, smoking, country

ALT, alanine aminotransferase; BMI, body mass index; CD4, cluster of differentiation 4; DBP, diastolic blood pressure; HDL, high-density lipoprotein cholesterol; FH of DM, family history of diabetes mellitus; FPG, fasting plasma glucose; HIV, human immunodeficiency virus; MAP, mean arterial blood pressure; SBP, systolic blood pressure; TG, triglycerides; PA, physical activity.

^a^Including age, BMI, waist circumferences, PA, vegetable intake, past history of hyperglycemia, and taking anti-hypertensive agents ### including age, waist circumferences, height, hypertension, PA, smoking, meat intake, intake of whole-grain bread, coffee, and alcohol.

Table 2 describes the assessment of study quality. No study received a full score of 10. Also, there were some “no” responses to each of the items. Mean (standard deviation) score was 4.3 (1.6). Table 6 summarizes the T2DM-associated loci used in each study. The number of SNPs ranged from 2 to 65 (median, 14.0 SNPs). Table 7 indicates the rank of the genes that were commonly used in association with prevalent or incident T2DM. The top 10 genes were *CDKAL1*, *TCF7L2*, *CDKN*, *HHEX*, *IGFBP2*, *SLC30A8*, *KCNJ11*, *PPARG*, *FTO*, and *KCNQ1*. Total number of SNPs that were covered by at least one of the included studies was 116.

**Table 6.** Details of genes used in each included study that are associated with type 2 diabetes mellitus

First author	Year	Number of SNPs	
Oian^18^	2015	9	*CDKAL1, CDKN2A/2B, CENTD2, FTO, HHEX* (2 SNP*s*), *KCNQ1, SLC30A8, VEGFA*
Talmud^17^	2015	65	*ADAMTS9, ADCY5, ANK1, ANKRD55, AP3S2, BCAR1, BCL11A, C2CD4A, CCND2, CDC123/CAMK1D, CDKAL1, CDKN2A/B, CENTD2, CILP2, DGKB, DUSP8, FTO, GCC1, GCK, GCKR, GIPR, GLIS3, GRB14, HHEX/IDE, HMG20A, HMGA2, HNF1A, HNF1B, HNF4A, IGF2BP2, IRS1, JAZF1, KCNJ11, KCNK16, KCNQ1, KLF14, KLHDC5, MAEA, MC4R, MTNR1B, NOTCH2, PEPD, PPARG, PRC1, PROX1, PSMD6, PTPRD, RBMS1, SLC30A8, SPRY2, SRR, ST64GAL1, TCF7L2, THADA, TLE1, TLE4, TP53INP1, TSPAN8/LGR5, UBE2E2, VPS26A, WFS1, ZBED3, ZFAND3, ZFAND6, ZMIZ1*
Chen^21^	2014	19	*ADAMTS9-MAGI1, ANK1, BCL11A-EIF3FP3, C6orf57, CDC123-CAMK1D, CDKAL1, CDKN2A/2B* (2 SNP*s*), *FTO, HHEX-EPOC6, HNF4A, IGF2BP2* (2 SNP*s*), *IRS1-KIAA1486, KLF14-FLJ43663, PTPRD, THADA, TSPAN8-LGR5, VPS26A*
Langenberg^20^	2014	49	*ADAMTS9, ADCY5, ANK1, ANKRD55, BCAR1, BCL11A, CCND2, CDC123-CAMK1D, CDKAL1, CDKN2A/2B, CENTD2, CILP2, DUSP9, DGKB, FTO, GCK, GCKR, GIPR, GRB14, HHEX-IDE, HMG20A, HMGA2, HNF1A, HNF1B, IGF2BP2, IRS1, KCNJ11, KCNQ1, KLF14, KLHDC5, MC4R, MTNR1B, NOTCH2, PPARG, PRC1, PROX1, SLC30A8, SPRY2, TCF7L2, THADA, TLE1, TLE4, TP53INP1, TSPAN8-LGR5, UBE2E2, WFS1, ZBED3, ZFAND6, ZMIZ1*
Imamura^25^	2014	10	*ANK1, C2CD4A/B, CDKAL1, CDKN2A/B, DUSP9, IGF2BP2, KCNQ1, MAEA, TCF7L2, UBE2E2*
Villegas^19^	2014	15	*ADAMTS9, CDC123-CAMK1D, CDKAL1, CDKN2A/2B, FTO, HHEX-IDE, IGF2BP2, JAZF1, KCNQ1, NOTCH2, PPARG, SLC30A8, TCF7L2, THADA, TSPAN8-LGR5*
Anand^26^	2013	16	*CDKAL1, CDKN2A/2B, FTO, GATAD2A, GCKR, HHEX-IDE, HNF1B, IGF2BP2, IRS1, KCNJ11, KCNQ1, MTNR1B, PPARG, SLC30A8, TCF7L2, WFS1*
Kalnina^27^	2013	2	*FTO, TMEM18*
Peters^28^	2013	3	*ADIPOQ* (3 SNP*s*)
Ramya^24^	2013	5	*ADIPOQ* (5 SNP*s*)
Robiou-du-Pont^23^	2013	24	*AIF1, BDNF* (2 SNP*s*), *CTNNBL1, ETV5, FAIM2, FTO* (2 SNP*s*), *GNPDA2, KCTD15, MAF, MC4R, MTCH2, NEGR1, NPC1, PCSK1* (2 SNP*s*), *PRL, PTER, SDCCAG8, SEC16B, SH2B1, TMEM18, TNKS*
Tam^22^	2013	14	*ADAMTS9, CDKAL1, CDKN2A/2B, HHEX, HNF1B, IGF2BP2, JAZF1, KCNJ11, KCNQ1, NOTCH2, SLC30A8, TCF7L2, TSPAN8-LGR5, WFS1*
Cauchi^29^	2012	13	*ADAMTS9, BCL11A, CDKAL1, CDKN2A/2B, FTO, GCK, HNF1A, IGF2BP2, KCNQ1, MC4R, TCF7L2, TP53INP1, WFS1*
Cooke^30^	2012	17	*ADAMTS9, CDKAL1, CDKN2A/2B, FTO, HHEX, HNF1B, IGF2BP2, JAZF1, KCNJ11, KCNQ1, NOTCH2, PPARG, SLC30A8, TCF7L2, THADA, TSPAN8-LGR5, WFS1*
Gamboa-Meiendez^31^	2012	21	*ADAMTS9, ARHGEF11, CDC123-CAMK1D, CDKAL1, CDKN2A/2B, FTO, HHEX* (2 SNP*s*), *IGF2BP2, JAZF1, KCNJ11, KCNQ1, NXPH1, NOTCH2, PPARG, RALGPS2, RORA, SLC30A8, TCF7L2, TSPAN8-LGR5, UBQLNL*
Iwata^32^	2012	14	*CDKAL1, CDKN2B, GCKR, HHEX, IGF2BP2, IRS1, KCNJ11, KCNQ1, PPARG, PRC1, PROX1, SLC30A8, TCF7L2, UBE2E2*
Long^33^	2012	29	*ADAMTS9, BCL11A, C2CD4A/B, CDKAL1* (2 SNP*s*), *CDKN2A/2B, CHCHD9, FTO, HHEX, HHEX-IDE, HMGA2, HNF1A, IGF2BP2* (2 SNP*s*), *JAZF1, KCNQ1* (3 SNP*s*), *KLF14, NOTCH2-ADAM30, RBMS1-ITGB6, SRR, TCF7L2, THADA, TSPAN8-LGR5, WFS1* (2 SNP*s*), *ZBED3, ZFAND6*
Vassy^34^	2012	38	*ADAMTS9, ADCY5, BCL11A, CDC123-CAMK1D, CDKAL1, CDKN2A/2B, CENTD2, DCD, DGKB-TMEM195, FTO, GCK, GCKR, HCCA2, HHEX, HMGA2, HNF1A, HNF1B, IGF2BP2, IRS1, JAZF1, KCNJ11, KCNQ1* (2 SNP*s*), *KLF14, MTNR1B, NOTCH2, PPARG, PRC1, PROX1, RBMS1-ITGB6, SLC30A8, TCF7L2, THADA, TLE4, TP53INP1, TSPAN8-LGR5, VEGFA, WFS1*
Vassy^35^	2012	38	*ADAMTS9, ADCY5, BCL11A, CDC123-CAMK1D, CDKAL1, CDKN2A/2B, CENTD2, DCD, DGKB-TMEM195, FTO, GCK, GCKR, HCCA2, HHEX, HMGA2, HNF1A, HNF1B, IGF2BP2, IRS1, JAZF1, KCNJ11, KCNQ1* (*2* SNP*s*), *KLF14, MTNR1B, NOTCH2, PPARG, PRC1, PROX1, RBMS1-ITGB6, SLC30A8, TCF7L2, THADA, TLE4, TP53INP1, TSPAN8-LGR5, VEGFA, WFS1*
Villegas^36^	2012	14	*CDC123-CAMK1D, CDKAL1* (*2* SNP*s*), *CDKN2A/2B, HHEX-IDE* (*2* SNP*s*), *HNF1B, IGF2BP2, KCNJ11, KCNK15, KCNQ1, SLC30A8, SPRY2, TP53INP1*
Yamakawa-Kobayashi^37^	2012	17	*ADAMTS9, CDC123, CDC24A, CDKAL1, CDKN2A/2B, FTO, HHEX, HNF1B, IGF2BP2, JAZF1, KCNJ11, KCNQ1, PPARG, SLC30A8, TCF7L2, TSPAN8, UBE2E2*
Li^38^	2011	12	*BDNF, ETV5, FAIM2, FTO, GNPDA2, KCTD15, MC4R, MTCH2, NEGR1, SEC16B, SH2Bi, TMEM18*
^*^Martinez-Gomez^39^	2011	5	*CaPN10, IRS1, PPARG, TCF7L2* (2 SNP*s*)
Rees^40^	2011	28	*ADAMTS9, BCL11A, CDC123-CAMK1D, CDKAL1, CDKN2A/2B, CENTD2, CHCHD9, DUSP9, FTO, HHEX-IDE, HNF1A, IGF2BP2, IRS1, JAZF1, KCNJ11, KCNQ1* (2 SNP*s*), *KLF14, NOTCH2, PPARG, PRC1, SLC30A8, TCF7L2, THADA, TP53INP1, TSPAN8-LGR5, WFS1, ZBED3*
Tabara^41^	2011	10	*CDKAL1, CDKN2A/2B, GCKR, HHEX, IGF2BP2, KCNJ11, KCNQ1, PPARG, SLC30A8, TCF7L2*
Uusitupa^42^	2011	19	*ADAMTS9, CDC123, CDKAL1, CDKN2B, FTO, HHEX, HNF1B, IGF2BP2, JAZF1, KCNJ11, KCNQ1, MTNR1B, NOTCH2, PPARG, SLC30A8, TCF7L2, THADA, TSPAN8, WFS1*
Fontaine-Bisson^43^	2010	17	*CDC123-CAMK1D, CDKN2A/2B, DCD, EXT2, HHEX* (2 SNPs), *HNF1B, KCNJ11, LOC387761, NOTCH2, PPARG, SLC30A8, TCF7L2, THADA, TSPAN8-LGR5, VEGFA, WFS1*
Qi^44^	2010	17	*ADAMTS9, CDC123-CAMK1D, CDKAL1, CDKN2A/2B, GCKR, HHEX, HNF1B, IGF2BP2, JAZF1, KCNJ11, KCNQ1, MTNR1B, PPARG, SLC30A8, TCF7L2, TSPAN-LGR5, WFS1*
Rotger^45^	2010	4	*FTO, KCNJ11, TCF7L2, TSPAN-LGR5*
Wang^46^	2010	20	*ADAMTS9, CDC123, CDKAL1, CDKN2A/2B, FTO, HHEX, HNF1B, IGF2BP2, JAZF1, KCNJ11, KCNQ1, LOC387761, MTNR1B, NOTCH2, PPARG, SLC30A8, TCF7L2, THADA, TSPAN8, WFS1*
Waters^47^	2010	19	*ADAMTS9, CDC123, CDKAL1, CDKN2A/2B, FTO, HHEX, HNF1B, IGF2BP2, JAZF1, KCNJ11, *(2 SNP*s*), *NOTCH2, PPARG, SLC30A8, TCF7L2, THADA, TSPAN8, WFS1*
Xu^48^	2010	4	*CDKAL1, CDKN2A/2B, KCNQ1, SLC30A8*
Cornelis^49^	2009	10	*CDKAL 1, CDKN2A/2B* (2 SNP*s*), *HHEX, IGF2BP2, KCNJ11, PPARG, SLC30A8, TCF7L2, WFS1*
Hu^50^	2009	11	*CDKAL1, CDKN2A/2B, FTO, HNF1B, IDE-KIF11-HHEX, IGF2BP2, KCNJ11, KCNQ1, PPARG, SLC30A8, WFS1*
Lin^51^	2009	15	*ADAMTS9, CDC123-CAMK1D, CDKAL1, CDKN2A/2B, FTO, HHEX-IDE, IGF2BP2, JAZF1, KCNJ11, NOTCH2, PPARG, TCF7L2, THADA, TSPAN-LGR5, WFS1*
Miyake^52^	2009	11	*CDKAL1, CDKN2B, GCKR, HHEX, HNF1B, IGF2BP2, KCNJ11, KCNQ1, PPARG, SLC30A8, TCF7L2*
Nordman^53^	2009	3	*HHEX, IDE, TCF7L2*
Rong^54^	2009	7	*CDKAL1, CDKN2B, FTO, HHEX, IGF2BP2, SLC30A8, TCF7L2*
Schulze^55^	2009	20	*ADAMTS9, BCL11A, CDC123-CAMK1D, CDKAL1, CDKN2A/2B, DCD, FTO, HHEX, HNF1B, IGF2BP2, JAZF1, KCNJ11, NOTCH2, PPARG, SLC30A8, TCF7L2, THADA, TSPAN8-LGR5, VEGFA, WFS1*
Cauchi^56^	2008	15	*CAMTA1, CDKAL1, CDKN2A/2B, CXCR4, EXT2, HHEX, IGF2BP2, KCTD12, LDLR, LOC387761,*
Lyssenko^57^	2008	11	*CDKAL1, FTO, HHEX, IGF2BP2, JAZF1, KCNJ11, NOTCH2, PPARG, SLC30A8, TCF7L2WFS1*
Meigs^58^	2008	18	*ADAMTS9, BCL11A, CDC123-CAMK1D, CDKAL1, CDKN2A/2B, DCD, HHEX, IGF2BP2, INS, JAZF1, KCNJ11, NOTCH2, PPARG, SLC30A8, TCF7L2, THADA, TSPAN8-LGR5, VEGFA*
Vaxillaire^59^	2008	3	*GCK, IL6, TCF7L2*
Scott^60^	2006	3	*KCNJ11, PPARG, TCF7L2*
Hansen^61^	2005	2	*KCNJ11, PPARG*
^*^Zacharova^62^	2005	2	*ADIPOQ* (2 SNPs)

SNP, single nucleotide polymorphism.

^*^The 2 studies used a dominant model.

The other studies used an additive model.

**Table 7.** Rank of the number of genes that were used to examine the association with the prevalence or incidence of type 2 diabetes mellitus

Number of studies	Genes
34	*CDKAL1, TCF7L2*
33	*CDKN, HHEX*
31	*IGFBP2*
29	*SLC30A8*
28	*KCNJ11*
27	*PPARγ*
25	*FTO, KCNQ1*
21	*TCF1B, TSPAN8*
20	*ADAMST9, WFS1*
19	*JAZF1*
18	*NOTCH2*
17	*CDC123*
16	*THADA*
10	*BCL11A*
9	*GCKR, IRS1*
8	*HNF1A*
7	*KLF14, MTNR1B, TP53INP1*
6	*CENTD2, GCK, PRC1, VEGFA*
5	*DCD, HMGA2, MC4R, PROX1, UBE2E2*
4	*ADCY5, ANK1, DGKB, TLE4, RBMS1-ITGB6, ZBED3*
3	*ADIPOQ, C2CD4A/B, DUSP9, LOC387761, SPRY2, ZFAND6*
2	*ANKRD55, BCAR1, BDNF, CCND2, CHCHD9, CILP2, ETV5, EXT2, FAIM2, GIPR, GNPDA2, GRB14, HCCA2, HMG20A, KCTD15, KLHDC5, MAEA, MTCH2, NEGR1, PTPRD, SEC16B, SH2B1, SRR, TLE1, VPS26A, ZMIZ1*
1	*AIF1, AMTA1, AP3S2, ARHGEF11, C6orf57, CaPN10, CDC24A, CTNNBL1, CXCR4, DUSP8, EXT2, GATAD2A, GCC1, GLIS3, HNF4A, IL6, INS, KCNK15, KCNK16, KCTD12, IDE, LDLR, LOC646279, MAF, MMP26, NXPH1, NGN3, NPC1, PCSK1, PEPD, PRC1, PRL, PROX1, PSMD6, PTER, RALGPS2, RORA, SDCCAG8, ST64GAL1, TMEM18, TNKS, UBQLNL, ZFAND3*

### Cross-sectional studies

**Figure 2.**
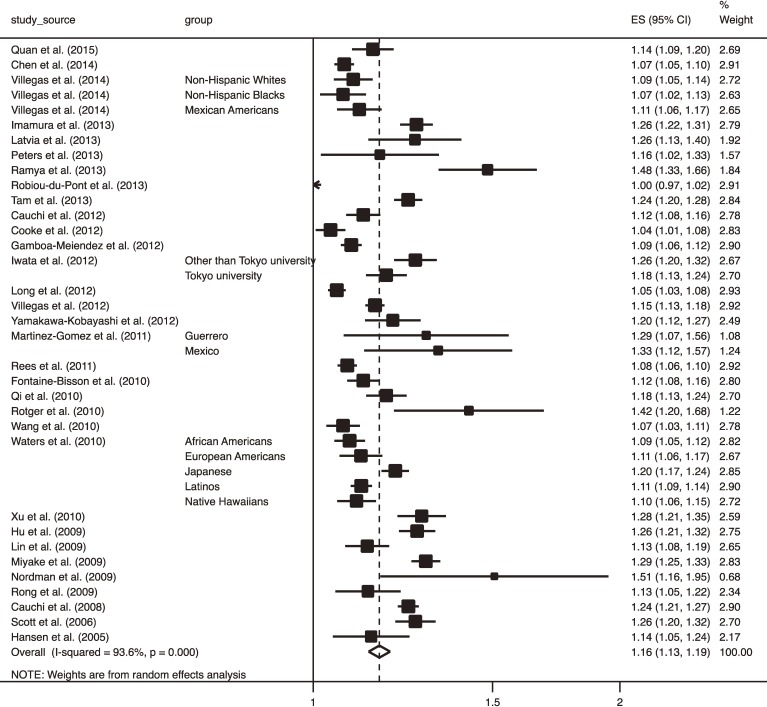
Forest plot of odds ratio (OR) with 95% confidence interval (CI) for risk of type 2 diabetes mellitus with 95% CI for 1 increment in risk alleles carried in cross-sectional studies. The OR in each study and the overall OR are indicated by squares and a diamond, respectively. Horizontal lines indicate the range of the 95% CI. The area of each square is proportional to the study weight expressed as the inverse of the square of standard error based on a random-effects model.

Among the 46 included studies, 32 studies^18^^,^^19^^,^^21^^–^^25^^,^^27^^–^^33^^,^^36^^,^^37^^,^^39^^,^^40^^,^^43^^–^^48^^,^^50^^–^^54^^,^^56^^,^^60^^,^^61^ that included 145,162 participants and 57,985 cases used a cross-sectional design. Mean age and BMI ranged from 33.7–68.8 years and from 23.2–36.9 kg/m^2^, respectively. The median proportion of men was 47%. Figure 2 shows a forest plot of ORs for T2DM, with a 95% confidence interval (CI), for 1-ΔRA. Overall, the OR for T2DM was highly significant (OR 1.16; 95% CI, 1.13–1.19; *P* < 0.001). However, there was highly significant between-study heterogeneity in the magnitude of ORs for T2DM (I-squared = 93.6%, *P* < 0.001). Publication bias was statistically suggested using both Begg’s and Egger’s tests (*P* = 0.03 and *P* = 0.01, respectively). Adjustment for publication bias using the trim and fill method slightly attenuated the T2DM risk but it remained highly significant (OR 1.15; 95% CI, 1.12–1.18; *P* < 0.001).

Table 8 shows the results of the stratified analysis of T2DM risk for 1-ΔRA using several pre-specified study characteristics. The OR for 1-ΔRA was consistently significant throughout any strata within individual subheadings. However, studies using 10 or more SNPs for T2DM screening revealed a smaller OR than those using less than 10 SNPs (pooled OR 1.14; 95% CI, 1.11–1.17 vs OR 1.25; 95% CI, 1.19–1.31; *P* = 0.002). The strength of the association was not significantly influenced by whether the OR was adjusted for BMI (*P* = 0.82). A significantly stronger association with T2DM risk was observed in studies of participants having a relatively lower mean BMI (<27 kg/m^2^ vs ≥27 kg/m^2^; *P* = 0.03) while a significantly weaker association was observed in studies that were women-dominant (*P* = 0.04). A negative association between mean BMI and T2DM risk for 1-ΔRA was also observed by entering the mean BMI as a continuous variable, predicting ORs of 1.18 and 1.11 for mean BMIs of 30 kg/m^2^ and 25 kg/m^2^, respectively. The pooling of studies that were considered to primarily represent Asian ethnicity, which were conducted in Japan, China, India, Singapore, or Pakistan, or targeted for Japanese-Americans, resulted in a significantly larger OR than the pooling of the other studies (*P* = 0.01). Also, excluding studies conducted in Western countries, including the United States, Mexico, Latvia, the United Kingdom, Sweden, Switzerland, France, Canada, or Denmark, resulted in a smaller OR compared with studies conducted in those Western countries (*P* = 0.02). Studies that excluded individuals with IGT from the non-T2DM cases revealed a larger OR compared with studies that did not (*P* = 0.002), while studies that excluded individuals with IFG did not influence the magnitude of the OR. Analysis was not influenced by whether or not OGTT was used for T2DM screening (*P* = 0.17).

**Table 8.** Stratified meta-analysis of eligible cross-sectional studies by several study items related to study characteristics for the pooled odds ratio (OR) for type 2 diabetes mellitus (DM) per 1 increase in risk alleles carried in relation to single nucleotide polymorphism (SNP)

Item		Number of data	OR (95% CI)	Q statistics	I-squared	*P* value of heterogeneity	Meta-regression $
Total		40	1.16 (1.13–1.19)	613.3	93.6%	<0.001	

Number of SNPs	<10	12	1.25 (1.11–1.31)	37.8	70.9%	<0.001	
	≥10	28	1.14 (1.11–1.17)	522.1	94.8%	<0.001	0.002

Mean age	<55 years	16	1.17 (1.11–1.22)	191.4	92.2%	<0.001	
	≥55 years	24	1.16 (1.13–1.20)	395.5	94.2%	<0.001	0.95

Proportion of men	<50%	26	1.14 (1.11–1.17)	361.5	93.1%	<0.001	
	≥50%	13	1.21 (1.16–1.26)	101.6	88.2%	<0.001	0.04#
	N/A	1	1.13 (1.05–1.22)	—	—	—	—

Country	Western	24	1.13 (1.10–1.16)	307.0	92.5%	<0.001	
	Non-Western	16	1.20 (1.16–1.25)	213.7	93.0%	<0.001	0.01

Dominant ethnic group	Asian	16	1.20 (1.16–1.25)	233.4	93.6%	<0.001	
	Non-Asian	24	1.13 (1.10–1.16)	273.4	91.6%	<0.001	0.01

Mean BMI^*^	<27 kg/m^2^	22	1.19 (1.15–1.24)	448.4	95.3%	<0.001	
	≥27 kg/m^2^	18	1.11 (1.09–1.14)	88.3	80.7%	<0.001	0.03

Adjustment for BMI	Yes	31	1.16 (1.13–1.20)	535.5	94.4%	<0.001	
	No	9	1.15 (1.10–1.21)	66	87.9%	<0.001	0.82

Using OGTT to diagnose DM	Yes	14	1.19 (1.14–1.25)	205.1	93.7%	<0.001	
	No	26	1.15 (1.11–1.18)	389.0	93.6%	<0.001	0.17

Excluding IGT from non-cases	Yes	7	1.28 (1.22–1.34)	15.4	31.0%	0.009	
	No	33	1.14 (1.12–1.17)	508.5	93.5%	<0.001	0.002

Excluding IFG from non-cases	Yes	13	1.18 (1.13–1.23)	189.6	93.7%	<0.001	
	No	27	1.15 (1.12–1.19)	406.5	93.6%	<0.001	0.42

BMI, body mass index; CI, confidence interval; DM, diabetes mellitus; IFG, impaired fasting glucose; IGT, impaired glucose tolerance; OGTT, oral glucose tolerance test; OR, odds ratio; SNPs, single nucleotide polymorphisms.

Table 9 shows the results of the multivariate meta-regression analysis that simultaneously entered several of the study characteristics used in the stratified analyses as explanatory variables. Consistent with the results of the stratified analyses that were previously described (ie, univariate analyses), studies that had a women-dominant population and were examined in Western countries revealed a smaller OR for T2DM for 1-ΔRA (*P* = 0.005 and *P* = 0.02, respectively) while studies that primarily targeted those of Asian ethnicity revealed a larger OR (*P* = 0.02). Unlike the results of the univariate analysis, neither the mean BMI, country where the individual study was conducted (Western or non-Western), nor the ethnic group (Asian or non-Asian) influenced the magnitude of the OR. However, in the multivariate analysis, the influence of the mean BMI became non-significant.

**Table 9.** Univariate and multivariate meta-regression analyses of eligible cross-sectional studies for odds ratio for type 2 diabetes mellitus (T2DM) for 1 increment in risk alleles carried in relation to diabetes-associated single nucleotide polymorphisms (SNPs) by characteristics of study design^a^

Variable	Univariate^b^ *N* = 40			Multivariate 1 *N* = 39^c^			Multivariate 2 *N* = 39^c^		
		
	Coefficient^d^	SE	*P*	Coefficient	SE	*P*	Coefficient	SE	*P*
No. SNPs ≥10	−0.094	0.029	0.002	−0.077	0.033	0.03	−0.084	0.033	0.02
Western country	−0.062	0.025	0.02	−0.060	0.025	0.02	^e^		
Asian ethnicity	0.065	0.025	0.01	^e^			0.081	0.031	0.02
Mean age ≥55 years	−0.002	0.028	0.95	−0.003	0.021	0.90	−0.019	0.023	0.42
Women-dominant^c^	−0.061	0.028	0.04	−0.065	0.022	0.005	−0.064	0.022	0.006
BMI ≥27 kg/m^2^	−0.057	0.025	0.03	−0.005	0.025	0.84	0.018	0.027	0.56
Adjustment for BMI	0.009	0.033	0.79	0.024	0.028	0.40	0.024	0.028	0.39
Using OGTT to diagnose DM	0.039	0.028	0.17	−0.004	0.026	0.88	−0.017	0.027	0.53
Excluding IGT from noncases	0.115	0.035	0.002	0.074	0.046	0.12	0.074	0.046	0.12
Excluding IFG from noncases	0.023	0.028	0.42	−0.019	0.023	0.42	−0.010	0.023	0.66

R-squared					64.4%			61.4%	
F-test					F(9,29) = 4.60	*P* < 0.001		F(9,29) = 4.70	*P* < 0.001

Abbreviations: Same as in Table 8.

^a^Logarithm of odds ratio for T2DM was a dependent variable, and each study characteristic was entered as an explanatory variable.

^b^Fundamentally, results were consistent with the stratified analysis.

^c^*N* = 39 because one study^54^ in which the proportion of men was not available was excluded.

^d^Positive value of the coefficient means that the OR is higher when the answer to each variable is “Yes” compared with a “No” answer. The same interpretation of the results is applied to Table 10, Table 12, and Table 13.

^e^Western country and Asian ethnicity was not entered simultaneously because of collinearlity.

Table 10 shows the results of univariate and multivariate meta-regression analyses according to whether each of the top 10 previously mentioned genes were used or not. In both univariate and multivariate analyses, the FTO gene was associated with a lower OR for T2DM. In the multivariate analysis, the 10 genes explained 59.9% of the variance in the log OR (*P* = 0.04).

**Table 10.** Univariate and multivariate meta-regression analyses of eligible cross-sectional studies for odds ratio for type 2 diabetes mellitus (T2DM) for 1 increment in risk alleles carried in relation to diabetes-associated single nucleotide polymorphisms (SNPs) by whether or not each of the top 10 of commonly used genes in this meta-analysis was examined^a^

Genes	Univariate *N* = 40			Multivariate *N* = 40		
	
	coefficient	SE	*P*	coefficient	SE	*P*
*CDKAL1*	−0.073	0.033	0.03	−0.061	0.069	0.38
*TCF7L2*	−0.016	0.031	0.60	0.015	0.035	0.65
*CDKN*	−0.073	0.033	0.03	^b^		
*HHEX*	−0.067	0.029	0.03	−0.025	0.056	0.65
*IGFBP2*	−0.061	0.029	0.04	0.010	0.052	0.84
*SLC30A8*	−0.029	0.029	0.03	0.026	0.047	0.58
*KCNJ11*	−0.004	0.027	0.89	0.008	0.033	0.81
*PPARγ*	−0.030	0.027	0.28	−0.025	0.037	0.51
*FTO*	−0.101	0.021	<0.001	−0.087	0.027	0.003
*KCNQ1*	−0.037	0.028	0.20	0.011	0.041	0.08

R-squared					59.9%	
F-test					F(9,30) = 2.39	*P* = 0.04

SE, standard error.

^a^Logarithm of odds ratio for diabetes mellitus was a dependent variable, and each study characteristic was entered as an explanatory variable.

^b^*CDKN* could not be entered simultaneously because of collinearlity.

### Longitudinal studies

**Figure 3.**
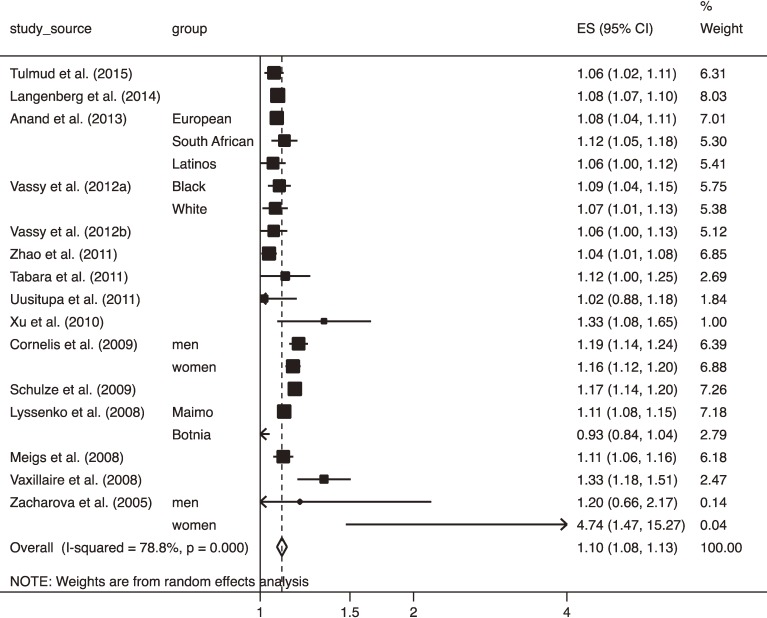
Forest plot of odds ratio (OR) with 95% confidence interval (CI) for risk of type 2 diabetes mellitus with 95% CI for 1 increment in risk alleles carried in longitudinal studies. The OR in each study and the overall OR are indicated in squares and a diamond, respectively. Horizontal lines indicate the range of the 95% CI. The area of each square is proportional to the study weight expressed as the inverse of the square of standard error based on a random-effects model.

Included were 15 longitudinal studies^17^^,^^20^^,^^26^^,^^34^^,^^35^^,^^38^^,^^41^^,^^42^^,^^48^^,^^49^^,^^55^^,^^57^^–^^59^^,^^62^ comprised of 104,203 participants, among which 17,666 participants developed T2DM. Figure 3 shows a forest plot of ORs of T2DM with 95% CI of T2DM for 1-ΔRA. The pooled OR for 1-ΔRA was 1.10 (95% CI, 1.08–1.13), which was significantly smaller than when cross-sectional studies were pooled (*P* = 0.04). Publication bias was detected using Egger’s test (*P* = 0.04) but not using Begg’s test (*P* = 0.40). However, the adjustment for publication bias resulted in no change in the overall estimate.

Two studies^26^^,^^62^ targeted exclusively IGT participants, while the other studies did not specify participants at high risk of T2DM according to IGT values. However, after excluding those studies, the overall OR was not influenced (OR 1.11; 95% CI, 1.08–1.14; *P* = 0.70). In the results of stratified and multivariate meta-regression analyses using several study characteristics, no variables that influenced T2DM risk could be detected, partly because these sensitivity analyses had insufficient statistical power due to the small number of datasets (Table 11 and Table 12). Nevertheless, studies using ≥10 SNPs revealed a smaller OR than those using <10 SNPs in the stratified analysis (pooled OR 1.10; 95% CI, 1.07–1.12 vs OR 1.34; 95% CI, 1.21–1.49; *P* = 0.005) (Table 11). Studies using 10 or more SNPs for T2DM screening revealed smaller ORs than those using less than 10 SNPs. Table 13 shows results of univariate and multivariate meta-regression analyses indicating whether each of the top commonly used genes was used for prediction of T2DM. Although none of the 10 genes significantly influenced the log OR of T2DM risk in both univariate and multivariate meta-regression analyses, multivariate analysis indicated that 67.5% of the variance in the log OR could be explained by whether or not each of the 10 genes was used (*P* = 0.006).

**Table 11.** Stratified meta-analysis of eligible longitudinal studies by several study items related to study characteristics for pooled odds ratio (OR) of type 2 diabetes mellitus (DM) per 1 increment in risk alleles carried in relation to single nucleotide polymorphism (SNP)

Item		Number of data	OR (95% CI)	Q statistics	I-squared	*P* value of heterogeneity	Meta-regression^d^
Total		21	1.10 (1.08–1.13)	94.5	78.8%	<0.001	

Number of SNPs	<10	4	1.34 (1.21–1.49)	4.6	35.2%	0.20	
	≥10	17	1.10 (1.07–1.12)	74.8	78.6%	<0.001	0.005

Mean age^b^	<50 years	9	1.11 (1.06–1.14)	28.6	72.0%	<0.001	
	≥50 years	11	1.11 (1.07–1.15)	60.2	83.4%	<0.001	0.95

Proportion of men	<50%	15	1.09 (1.06–1.12)	65.3	78.6%	<0.001	
	≥50%	6	1.14 (1.09–1.20)	21.3	76.6%	0.00	0.19

Country	Western	19	1.10 (1.08–1.13)	91.1	80.2%	<0.001	
	Non-Western	2	1.16 (1.05–1.29)	2.1	51.3%	0.15	0.40

Dominant ethnic Group	White	15	1.10 (1.08–1.13)	89.3	83.2%	<0.001	
	Non-White	5	1.10 (1.06–1.13)	5.2	23.1%	0.27	0.94

Mean BMI^a,b,c^	<25 kg/m^2^	7	1.12 (1.08–1.17)	15.6	61.5%	0.02	
	≥25 kg/m^2^	13	1.10 (1.07–1.13)	75.8	84.2%	<0.001	0.5

Adjustment for BMI	Yes	17	1.10 (1.08–1.13)	63.0	74.6%	<0.001	
	No	4	1.10 (1.05–1.16)	18.5	83.8%	<0.001	0.96

Using OGTT to diagnose DM	Yes	12	1.11 (1.07–1.15)	41.5	73.5%	<0.001	
	No	9	1.10 (1.06–1.13)	45.1	82.3%	<0.001	0.80

BMI, body mass index; OGTT, oral glucose tolerance test; IGT, impaired glucose tolerance; N/A, not applicable.

^a^Indicates mean BMI of study population at recruitment.

^b^One study^17^ did not present data on mean age or BMI.

^c^Indicates mean BMI at the beginning of follow-up.

^d^*P* value for difference in the magnitude of logarithm of odds ratio between strata was indicated.

**Table 12.** Univariate and multivariate meta-regression analysis of eligible longitudinal studies for odds ratio of type 2 diabetes mellitus for 1 increment in risk alleles in relation to diabetes-associated single nucleotide polymorphism (SNP)^a^ according to characteristics of study design^a^

Variable	Univariate^c^ *N* = 21			Multivariate 1 *N* = 20^b^			Multivariate 2 *N* = 20^b^		
		
	coefficient	SE	*P*	coefficient	SE	*P*	coefficient	SE	*P*
Number of SNPs ≥10 (Yes/No)	−0.206	0.066	0.005	−0.182	0.083	0.049	−0.186	0.082	0.04
Western country (Yes/No)	−0.067	0.078	0.40	−0.035	0.091	0.7	^d^		
White-dominant (Yes/No)	−0.003	0.039	0.94	^d^			0.002	0.04	0.97
Mean age ≥50 years (Yes/No)^b^	0.002	0.034	0.95	0.01	0.04	0.82	0.017	0.037	0.67
Women-dominant	−0.045	0.033	0.19	−0.052	0.042	0.24	−0.049	0.042	0.27
BMI ≥25 kg/m^2^ (Yes/No)	−0.025	0.036	0.50	0.01	0.048	0.84	0.000	0.043	1.00
Adjustment for BMI	0.002	0.038	0.96	−0.011	0.04	0.79	−0.007	0.042	0.88
Using OGTT to diagnose DM	0.008	0.032	0.80	−0.006	0.035	0.87	−0.004	0.035	0.91

R-squared					49.1%			47.5%	
F-test					F(7,12) = 1.39	*P* = 0.29		F(7,12) = 1.37	*P* = 0.30

BMI, body mass index; DM, diabetes mellitus; OGTT, oral glucose tolerance test; SE, standard error.

^a^Logarithm of odds ratio for diabetes mellitus was a dependent variable, and each study characteristic was entered as an explanatory variable.

^b^*N* = 20 because one study^17^ did not present data on mean age or BMI.

^c^Fundamentally, the results were consistent with the stratified analysis.

^d^Western country and Asian ethnicity was not entered simultaneously because of collinearlity.

**Table 13.** Univariate and multivariate meta-regression analyses of eligible longitudinal studies for odds ratio for type 2 diabetes mellitus (T2DM) for 1 increment in risk alleles carried in relation to diabetes-associated single nucleotide polymorphisms (SNPs) by whether or not each of the top 10 of the commonly used genes in this meta-analysis was examined^a^

Genes	Univariate *N* = 21			Multivariate *N* = 21		
	
	coefficient	SE	*P*	coefficient	SE	*P*
*CDKAL1*	−0.031	0.055	0.58	^b^		
*TCF7L2*	0.011	0.058	0.86	0.261	0.096	0.02
*CDKN*	−0.024	0.044	0.58	0.044	0.030	0.17
*HHEX*	−0.057	0.051	0.28	−0.486	0.190	0.02
*IGFBP2*	−0.057	0.051	0.28	^b^		
*SLC30A8*	−0.031	0.055	0.58	0.292	0.163	0.10
*KCNJ11*	−0.057	0.051	0.28	^b^		
*PPARγ*	−0.057	0.051	0.28	^b^		
*FTO*	−0.057	0.03	0.08	0.014	0.022	0.59
*KCNQ1*	−0.042	0.028	0.16	−0.074	0.022	0.004

R-squared					67.5%	
F-test					F(6,14) = 5.09	*P* = 0.006

SE, standard error.

^a^Logarithm of odds ratio for diabetes mellitus was a dependent variable, and each study characteristic was entered as an explanatory variable.

^b^The 4 genes could not be entered simultaneously because of collinearlity.

## DISCUSSION

The current meta-analysis indicated that 1-ΔRA in T2DM-associated SNPs was associated with a modest risk of prevalent or incident T2DM. A previous meta-analysis^6^ indicated that the pooled RR of incident T2DM was 1.87 for an increment of 4.3 kg/m^2^ in the BMI. If the magnitude of T2DM risk is compared between 1-ΔRA and 1-ΔBMI, 1-ΔRA corresponds to only 0.58-ΔBMI (Δ1.7 kg, if body height is 1.7 meters), assuming that the cumulative incidence rate of T2DM in the referent is p_0_ = 10% and the OR is transformed into an RR using the following formula: $\text{RR}=\frac{\text{OR}}{(1-{\text{p}}_{0})+{\text{p}}_{0}\times \text{OR}}$.^8^ This estimation was made by the following calculations:

$$\begin{array}{l} \frac{1.10}{(1-0.1)+0.1\times 1.10}=1.089\\ \text{log}(1.089)=0.085\\ \frac{\text{log}(1.87)}{4.3}=0.146\\ \frac{0.085}{0.146}=0.58 \end{array}$$

In other words, having 1 RA was equivalent to losing less than 2 kg body weight.

Assuming the monotonicity between the number of RAs carried and T2DM risk, the magnitude of disease risk will endlessly expand with increases in the RAs carried, even if the effect size of each SNP is modest. Therefore, GWAS may be able to detect individuals at extremely high risk of T2DM if the number of the identified T2DM-associated SNPs is progressively increased. However, the magnitude of T2DM risk might reach a plateau, which was suggested from the result of the stratified analysis that indicated that studies using a larger number of SNPs (10 or more) revealed a smaller OR of T2DM for 1-ΔRA compared with those using a smaller number of SNPs. Although the observed higher risk when using a smaller number of genes could be spurious due to the winner’s curse effect (eg, T2DM risk would be higher when using only the top two genes that were strongly associated with susceptibility to T2DM than when using the top 10 genes), this result reflects the fact that the T2DM-susceptible loci were detected in order of descending effect size related to T2DM risk among all proposed loci.^65^ Of note, although the magnitude of genetic T2DM risk was modest, this did not mean that the risk was ignorable from the result of the stratified analysis that indicated that the association of cumulative RAs with T2DM risk was not weakened after adjusting the T2DM risk for BMI. This result suggested that the genetic T2DM risk existed independently of obesity, which is well known as one of the greatest risk factors for T2DM.

The current meta-analysis indicated a large heterogeneity in the magnitude of the T2DM risk for cumulative RAs, which was in a large part explained by the choice of the T2DM-susceptible genes. This suggested that the magnitude of T2DM risk depended on what genes were used. In particular, the cross-sectional studies that used the *FTO* gene revealed a lower OR compared with those that did not use it, which suggested that it is unnecessary to examine the *FTO* gene if clinical risk factors for T2DM were simultaneously assessed. *FTO* is one of the most well-known of the obesity-associated genes.^66^ Considering that the OR for T2DM was adjusted for obesity in most of the studies included in this meta-analysis, the T2DM risk associated with the *FTO* gene could have been masked by that associated with obesity. Although the magnitude of T2DM risk was not influenced by whether or not *FTO* was used in the longitudinal studies, the inconsistency in the results between the meta-analysis of cross-sectional studies and that of longitudinal studies could be explained by differences in the exposure to risk factors (ie, participants in longitudinal studies were recruited before they became obese, although those in cross-sectional studies had already become obese at recruitment). Despite the heterogeneity in the choice of the T2DM-susceptible genes, the forest plots shown in Figure 2 and Figure 3 indicate that the OR for carrying 1-ΔRA was within 1.0–1.3. This value corresponds to a modest effect size.^4^ Therefore, it is unlikely that heterogeneity in the genes among studies changed the main conclusion of this study. Nevertheless, the heterogeneity in the magnitude of T2DM risk due to the heterogeneity among the used genes urges us interpret the results with caution.

The current stratified meta-analysis of cross-sectional studies indicated that a larger magnitude of T2DM risk in relation to cumulative RAs was observed in studies with a proportion of men 50% or greater compared with women-dominant studies, suggesting that the magnitude of the genetic association with T2DM was larger in men than in women. Although evidence for gender-related differences in genetic associations has been insufficient,^67^ some major longitudinal studies suggested that the environmental contribution to T2DM risk differed by gender. For example, regarding moderate physical activity, which has been established to reduce T2DM risk,^68^ the MONICA/KORA Augsburg Cohort study suggested that leisure time physical activity was effective in preventing T2DM, especially in women.^69^ In the intensive lifestyle modification group in the Diabetes Prevention Program, the effect in preventing incident T2DM did not differ by sex, although men lost significantly more body weight and increased physical activity more than did women.^70^ The relative contribution of genetic risk to incident T2DM could be smaller in women than in men. Further studies would need to analyze the T2DM risk in relation to genetic risk by gender to clarify the gender difference in the relative contribution of genetic profiles. Of note, this suggestion was contradictory to the finding in one of the included studies in this meta-analysis, which indicated that carrying RAs was associated with T2DM in women but not in men.^62^ However, this study focused on *ADIPOQ* among the established T2DM-susceptible genes and limited the subjects to those who already had IGT at cohort entry. *ADIPOQ* might play a role in the determination of the severity of glucose tolerance, especially in women, although the majority of SNPs used for T2DM screening was associated with discrimination of glucose intolerance from normal glucose tolerance.

Current sensitivity analyses indicated that there was a stronger association between cumulative RAs and T2DM risk in study populations with lower mean BMI. This finding was supported by previous studies that stratified the analyses by BMI, although these data could not be included in this meta-analysis because characteristics other than BMI in each subgroup were not presented.^20^^,^^25^ However, ethnicity or geographic region, rather than obesity, might affect the strength of the association between cumulative RAs and T2DM risk, considering that the prevalence of overweight and obesity in Asia is relatively low compared with Western populations.^71^ To our knowledge, no studies have investigated the difference in the magnitude of T2DM risk by ethnicity using two or more SNPs, although a difference in the attributable risk of a certain T2DM-susceptible SNP has been suggested.^72^ Whether an included study was targeted on an Asian-dominant ethnicity or whether it was conducted in Western countries affected the magnitude of T2DM risk for an increment in the number of RAs carried in both univariate and multivariate meta-regression analyses. However, modification of the magnitude of T2DM risk by the mean BMI that was shown in univariate analyses disappeared in the multivariate analyses. It could be interpreted that the contribution of genes to T2DM risk could be affected by ethnicity rather than physical background. Unfortunately, when applied to the included studies in the current meta-analysis, this hypothesis was directly elucidated by only one study, which compared the magnitude of T2DM risk between Asian and non-Asian ethnic groups.^47^ Further studies are needed to confirm the characteristics of individuals or populations that are susceptible to genetic T2DM risk, including determinations of whether the characteristic was the extent of obesity or ethnicity.

The result of the current stratified meta-analyses might reflect characteristics regarding metabolic traits of persons at high genetic risk of T2DM: a higher OR was observed in studies that excluded subjects without T2DM who had IGT compared with studies that did not exclude subjects with IGT, but no difference in ORs was observed according to whether or not subjects with IFG were excluded, which meant that inclusion of subjects with IGT in the non-case group weakened the association between cumulative RA and T2DM risk. A previous study reported that the genetic risk score was reported to be cross-sectionally associated with the risk of having IGT but not IFG using nine genes (*FTO, HHEX, KCNJ11, KCNQ1, MTNR1B, PPARG, SLC30A8, TCF7L2, WFS1*), which were established to be major loci associated with the risk of T2DM.^73^ When applying this finding to this meta-analysis, seven of these nine genes were ranked in the top 10 genes that frequently appeared in the included studies in this meta-analysis. Major T2DM-associated genes that were discovered in the current GWAS might have a stronger association with glucose intolerance than T2DM itself. Considering that T2DM is a partly a result of progression of glucose intolerance, incident T2DM could be prevented by non-genetic factors, although mild glucose tolerance was inevitably regulated by genes. This suggestion was supported by evidence for the benefit of lifestyle interventions in reducing the risk of progression from IGT to T2DM.^74^

Several limitations should be addressed. First, this meta-analysis had to assume the log-linearity between the number of RAs carried and the observed T2DM risk. Second, this meta-analysis assumed that each SNP within one study would equally contribute to the risk of T2DM, although the OR for each SNP varied. However, it was reported that the difference in the discriminative power between using an unweighted and weighted genetic risk score was not significant^43^ or was modest.^51^ Third, as previously mentioned, the genes that were used in the examination of the association with prevalence and incidence of T2DM were too heterogeneous to analyze by the combination of the used genes. Nevertheless, the genes used for genetic risk should be the same across the studies. Fourth, this meta-analysis also did not consider heterogeneity in covariates for which each study adjusted T2DM risk. To minimize this heterogeneity, analyses were limited to the ORs that were adjusted for at least two of three covariates: age, gender, and BMI. In addition, sensitivity analyses confirmed that the magnitude of T2DM risk was not influenced by whether these ORs were adjusted for BMI. Therefore, the current meta-analysis could confirm that the genetic T2DM risk was independent of age, gender, and obesity. Although these are well known to be major classic risk factors for T2DM, it needs to be emphasized that other residual confounders could not be ruled out.

### Conclusions

The current meta-analysis indicated that carrying one RA in T2DM-associated SNPs was associated with a modest risk of prevalent or incident T2DM as far as the SNPs discovered in GWAS were concerned, although the heterogeneity in the genes examined among studies indicates that we should interpret the results with caution.
